# Experiences from the Department of Infectious Disease Epidemiology at Robert Koch Institute

**DOI:** 10.25646/6506

**Published:** 2020-06-04

**Authors:** Thomas Harder

**Affiliations:** Robert Koch Institute, Berlin, Department of Infectious Disease Epidemiology

At Robert Koch Institute’s Department of Infectious Disease Epidemiology, approaches for applying the principles of evidence-based public health (EBPH) have been particularly successfully implemented in the Immunization Unit and in the Unit for Healthcare-associated Infections, Surveillance of Antibiotic Resistance and Consumption. Furthermore, both units participated in the international Project on a Framework for Rating Evidence in Public Health (PRECEPT) which has been funded by the European Centre for Disease Prevention and Control (ECDC).

The Immunization Unit hosts the Executive Secretariat of the German Standing Committee on Vaccination (STIKO). In 2011, STIKO has adopted a new Standard Operating Procedure (SOP) for the development of vaccination recommendations. Main aim was to provide a methodological framework to guide this process by applying the principles of EBPH. The SOP comprises ten consecutive steps, starting with a prioritization process of relevant topics, and ending with the publication of the recommendation and a related paper on the scientific background. Following the principles of EBPH, systematic reviews and meta-analyses are performed to address key issues. The methodology of the Grading of Recommendations Assessment, Development and Evaluation (GRADE) working group is a core element of the SOP and is used to assess the quality of evidence on vaccine efficacy/effectiveness and safety, including population effects. In addition, STIKO has started to use Evidence-to-Decision Tables to support the decision-making process. Since 2012, ten recommendations on new vaccines or revisions of previous recommendations have been successfully developed using the SOP, accompanied by publication of background papers and of the respective systematic reviews in peer-reviewed journals [1–3].

At the Unit for Healthcare-associated Infections, Surveillance of Antibiotic Resistance and Consumption, the EBPH approach has been used to perform systematic reviews on the burden of healthcare-associated infections [4]. As an example, in an ECDC-funded project the long-term consequences of health-care-acquired neonatal sepsis for the neurological development of preterm infants were assessed by meta-analysis [5]. Furthermore, a systematic review addressed the prognostic value of neonatal surface screening for gram-negative bacteria for sepsis prediction [6].

The latter review was part of the PRECEPT project. PRECEPT defined a methodology for evaluating and grading evidence in public health, with a particular focus on infectious disease epidemiology, prevention and control, taking different domains and question types into consideration [7]. The methodology rates evidence in four domains: disease burden, risk factors, diagnostics and intervention. The framework has four steps going from overarching questions to an evidence statement. In step 1, approaches for identifying key areas and developing specific questions are described. In step 2, methodological guidance for conducting systematic reviews is provided. In step 3, a standardized evidence-grading scheme using the GRADE methodology is provided. Step 4 consists of preparing a narrative evidence summary. The development of the approach was accompanied by piloting studies as well as studies on method ological aspects such as choice of risk of bias tools and use of existing systematic reviews for development of new recommendations [8, 9]. Dissemination of the PRECEPT approach was supported by the development of an e-learning tool hosted by ECDC.
